# Diabetes-induced osteoarthritis: role of hyperglycemia in joint destruction

**DOI:** 10.1186/1471-2474-16-S1-S1

**Published:** 2015-12-01

**Authors:** Alexandrina F Mendes, Susana C Rosa, Ana T Rufino, Madalena Ribeiro, Fernando Judas

**Affiliations:** 1 Faculty of Pharmacy, University of Coimbra, Coimbra, Portugal; 2 Center for Neuroscience and Cell Biology, University of Coimbra, Coimbra, Portugal; 3 University and Hospital Center of Coimbra, Coimbra, Portugal; 4 Faculty of Medicine, University of Coimbra, Coimbra, Portugal

## 

Recent epidemiologic and experimental data reinforced the concept that diabetes mellitus (DM) is an independent risk factor for osteoarthritis (OA). Besides a systemic inflammatory response that can affect joint tissues and contribute to OA pathogenesis, direct effects of hyperglycaemia have been shown to cause cell damage and induce inflammation by various mechanisms in several tissues associated to diabetic complications. Whether and how glucose directly affects joint tissues and cells is just beginning to be unraveled. Indirect effects of high glucose can result from enhanced formation of advanced glycation end products (AGEs) which accumulate in OA cartilage in an age-dependent manner and play a pro-inflammatory and pro-catabolic role mediated by activation of their specific receptor, RAGE, on chondrocytes and synovial cells. Some direct effects of high glucose have also been demonstrated, namely induction of IGF-1 resistance[1] and inhibition of dehydroascorbate transport which can compromise collagen synthesis[2]. Our studies have been aimed at determining whether and how hyperglycemia affects chondrocyte functions and contributes to OA development and progression. The results obtained showed that high and low glucose concentrations regulate the availability of facilitative glucose transporter (GLUT) isoforms and the glucose transport capacity of human chondrocytes. High glucose concentrations decrease the transport capacity and GLUT-1 protein content without affecting its mRNA levels, but this ability to adjust glucose transport capacity as a function of its availability is compromised in aged/OA chondrocytes leading to its intracellular accumulation[3]. The consequences of this are increased and prolonged ROS production[3] and expression of metalloproteinases (MMP)-1 and -13[4], IL-1β, TNF-α, inducible nitric oxide (NO) synthase (iNOS) and NO production, mediated by high glucose-induced NF-κB activation[5], as well as decreased responsiveness to TGF-β[4] and impaired autophagy[5]. High glucose is thus sufficient to induce an inflammatory and catabolic response in human OA chondrocytes. Furthermore, it potentiates pro-inflammatory effects of IL-1β, namely IL-6, cyclooxygenase 2 (Cox)-2, prostaglandin E2 (PGE2) and NO production[6]. The pro-inflammatory effects of high glucose in human chondrocytes and diabetic mice, namely induction of Cox-2, IL-6 and MMP-13 and production of PGE2, as well as decreased production of Collagen II, have also been shown to involve impairment of anti-inflammatory pathways, namely by decreasing PPAR-γ expression[7].

Elucidating how high glucose modulates joint tissue homeostasis will identify novel targets for development of innovative strategies both to identify diagnostic and prognostic biomarkers of OA and to effectively modify disease progression.
